# The Immediate Treatment of Puerperal Sepsis—II

**Published:** 1909-01-16

**Authors:** 


### Obstetrics.

### THE IMMEDIATE TREATMENT OF PUERPERAL SEPSIS—II.

In the last article on this subject the necessity for investigation of these cases was insisted upon, and the technique to be followed in making this investigation was described. Such investigation is to precede any form of treatment, so that washing out the uterus or other simple local treatment must be absolutely postponed until a thorough investigation has revealed the cause of the complaint. If the exploration of the uterus reveals retained placental fragments, membrane, or clots, and particularly if these are offensive and decomposing, their immediate removal will in most cases effect a cure. The sapræmic condition set up by decomposition in the uterus is the commonest form of puerperal sepsis and the easiest to cure, but it must be remembered that these offending materials are usually adherent to the uterus and no amount of washing out its cavity will then remove them. It is true, however, that washing out the uterus in such cases often produces a temporary fall in the temperature, and that other symptoms improve. But unless the decomposing material is removed the condition of the patient will soon become as bad as before, and further, the retained material becomes a culture medium for other organisms besides mere saprophytes; consequently we find that cases which begin with sapræmic symptoms sometimes go on to generalised blood infection (septicæmia), because these decomposing masses have incubated streptococci and other organisms, have possibly helped to raise their virulence, and finally the resistance of the individual has been overcome. Therefore the sooner retained decomposing material is removed from the uterus the more likely will a cure result, and, we believe, a certain percentage of septicæmias be prevented.

The best way to remove the retained materials is with the gloved fingers, gradually scraping them away from their attachments and allowing them to slide down the palm of the hand. In a fair proportion of cases all that is found is a knobbly condition of the placental site, in which the projecting knobs are dead tissues composed of fibrin and clot and are infected. These can be broken down carefully without injuring the uterine wall and improvement will result. After all obvious retained products have been removed the uterus is to be washed out with a freshly boiled double action tube of glass or metal. Plain boiled water at a temperature of 112° to 115° Fahr. is all that is required, but there is no objection to using an antiseptic, such as lysol in the strength of 1 or 2 drachms to the pint. It is an impossibility to sterilise the interior of the large flabby uterus; all that we can do is to wash out loose fragments.

This operation is often attended by free bleeding, owing to the relaxation of the uterus which occurs under the manipulation and the anæsthetic. A hypodermic injection of ergotin will usually secure firm contraction afterwards, and it is a good plan to keep the uterus contracted by drachm doses of the liquid extract of ergot three times a day for two or three weeks. The only general recommendation with regard to the operation is this, that the exploration must include every part of the uterine cavity; the angles and the fundus are the chief places where retained materials are found.

Vaginal douching after clearing out the uterus is quite unnecessary, and may indeed be harmful by introducing more sepsis. The bowels should be kept acting, and care must be taken that plenty of food is given. Alcohol is unnecessary.

Although the larger number of septic cases are thus cured, there are others in which the infection is more serious. The first principle in treating any septicæmia is to discover the infecting organisms; consequently in all these cases cultures should be made from the interior of the uterus. The reasons for this are two, to find out whether an antitoxic serum will possibly be of value, and to provide a culture from which a vaccine can be made if required. There is no great difficulty in getting a swab soaked in the uterine fluids provided it is not allowed to touch anything until it enters the cervix. As the patient is usually under an anæsthetic the best plan is to have ready a boiled Sim's speculum, a vulsellum forcep, and a sterilised swab in a plugged sterile tube. Having exposed the cervix with the speculum the anterior lip is seized with the vulsellum and pulled down so that the os uteri is visible and within reach. Blood and secretions are wiped with a piece of boiled cotton wool, and then the swab is passed about two inches into the cervical canal, and withdrawn carefully so as not to touch the vaginal walls. A great saving of time is effected by getting a swab at the first exploration of the uterus, and if the treatment of puerperal sepsis is to be attempted on modern lines by vaccines after Wright's method, time is all important. It takes an appreciable time—from thirty-six to forty-eight hours in most instances—to prepare a vaccine, although where one organism is particularly in evidence this time can sometimes be shortened.

*(To be continued.)*

